# Elevated Serum Total Bilirubin Level Is Associated with Poor Outcomes in Pediatric Patients with Sepsis-Associated Liver Injury

**DOI:** 10.1155/2018/4591729

**Published:** 2018-10-15

**Authors:** Yun Cui, Yijun Shan, Rongxin Chen, Chunxia Wang, Yucai Zhang

**Affiliations:** Department of Critical Care Medicine, Shanghai Children's Hospital, Shanghai Jiao Tong University, Shanghai 200040, China

## Abstract

**Aims:**

The aim of this study was to assess the prognostic value of the serum total bilirubin (TBIL) level in pediatric patients with sepsis-associated liver injury (SALI).

**Methods:**

We performed a retrospective study of patients with SALI admitted to the pediatric intensive care unit (PICU) in Shanghai Children's Hospital between December 2012 and December 2015. Serum TBIL concentration was determined within 72 h after PICU admission.

**Results:**

Seventy-two patients with SALI were included in this study. The overall mortality rate was 36.1% (26/72). The serum levels of TBIL of patients were significantly higher in the nonsurvivor group than the survivor group. *Cox* regression analysis indicated that the elevated serum TBIL level within 72 hours after admission was an independent risk factor of mortality in patients with SALI. Furthermore, the area under the receiver-operating characteristic (ROC) curve (AUC) for TBIL was 0.736 (95% confidence interval (CI): 0.614–0.858, *P*=0.001), in which the optimal cut-off value was 64.5 *μ*mol/L. The combined index named “TBIL” and “TBA” showed an AUC of 0.745 (0.626–0.865) for predicting the prognosis in patients with SALI. In addition, the Kaplan–Meier curve indicated that the 28-day survival rate was significantly lower in patients with higher serum TBIL levels (≥64.5 *μ*mol/L) or higher value of TBIL and TBA (≥−0.8902).

**Conclusions:**

Elevated serum TBIL level is associated with poor outcomes in pediatric SALI.

## 1. Introduction

Sepsis is defined as life-threatening organ dysfunction caused by a deregulated host response to infection [1]. Although the mortality rate for sepsis has declined over the past decade, it remains high in critically ill patients [2]. The liver plays a key role in regulation of host immune response to sepsis for clearing bacteria and toxins, which also causes inflammation, immunosuppression, and liver damage [3]. Liver dysfunction contributes to the metabolic derangements of critical illness and usually is associated with poor prognosis [3–5]. The incidence of sepsis-associated liver injury (SALI) in the adult is about 34.7% [6]. SALI is a significant predictive sign of poor prognosis in adult patients with sepsis [6]. Early detection and evaluation of SALI are important for improving the clinical outcomes. However, the prognostic prediction of pediatric SALI remains the little information available.

SALI is characterized by hyperbilirubinemia with or without the elevation of aminotransferases [7]. Hyperbilirubinemia is a risk factor for infection in the surgical intensive care unit (ICU) [8], and an admission bilirubin of greater than 2 mg/dL was independently associated with the development of sepsis-related acute respiratory distress syndrome (ARDS) and mortality in sepsis [9]. In addition, the elevated serum bilirubin level within 72 hours after admission is associated with an increased risk of mortality in the adult patients with severe sepsis and septic shock [10]. The prognostic value of hyperbilirubinemia in pediatric critically ill patients needs further study to verify.

We speculated that hyperbilirubinemia might play a specific prognostic role in pediatric patients with SALI. In present study, we compared the serum TBIL levels in survivors or nonsurvivors of the patients with SALI who admitted to the pediatric intensive care unit (PICU). The aim of our study was to assess the prognostic value of serum TBIL levels and the combined indexes of TBIL with other variables in pediatric patients with SALI.

## 2. Materials and Methods

### 2.1. Study Design

We performed a retrospective study of patients with SALI admitted to the PICU in Shanghai Children's Hospital between December 2012 and December 2015. Patients with sepsis were diagnosed based on the International Pediatric Sepsis consensus conference in 2005 [11] and Surviving Sepsis Campaign International Guidelines in 2012 [12]. All the enrolled patients had been diagnosed as SALI. The study was conducted in accordance with the ethical principles of the Declaration of Helsinki (and subsequent revisions) and to the current norm for observational studies. A requirement for the informed consent was waived. This study was approved by the Ethics Committee of Shanghai Children's Hospital (no. 2016R010-E02).

### 2.2. Patients

Patients aged 1 month to less than 18 years old who were diagnosed with SALI between December 2012 and December 2015 were screened for inclusion. Patients who died or were discharged within 72 hours after admission, or primary hepatobiliary involvement on admission, or inherited metabolic diseases, or toxicosis were excluded. Primary hepatobiliary involvement was defined as liver trauma, hepatitis, malignancy, and cholecystitis. SALI were diagnosed according to the criteria of International pediatric sepsis consensus conference [11]. SALI was defined by the following conditions: (1) TBIL ≥ 4.0 mg/dl or (2) ALT 2 times upper limit of normal for age. In all cases, anti-infection therapy was initiated when the patients met the criteria for sepsis. And drugs for keeping circulation stable and hepatic protection were used as standard management for all patients. The patients with kidney dysfunction or fluid overload were treated with continuous renal replacement therapy (CRRT). Mechanical ventilation was used in the patients with respiratory failure.

### 2.3. Data Collection and Definitions

All the data were retrieved from the electronic medical records. Variables were defined before data collection and entered in a standardized format during the data collection. The collected data included demographic data (such as age and gender), details of the initial clinical presentation on admission, Pediatric Risk of Mortality score (PRISM III) [13] within 24 hours following admission, positive bacterial cultures, comorbid diseases, the need for mechanical ventilation, CRRT, and 28-day mortality. Additional data about laboratory characteristics were the highest value within 72 hours after PICU admission obtained from the computerized hospital medical records.

The blood routine and biochemical determination were assessed in all patients. The indicators for liver dysfunction included alanine aminotransferase (ALT), *γ*-glutamyl transpeptidase (*γ*-GT), total bilirubin (TBIL), albumin (ALB), prothrombin time (PT), activated partial thromboplastin time (APTT), total bile acid (TBA), and international normalized ratio (INR). The values of mean arterial pressure (MAP), the ratio of the partial pressure of oxygen in the arterial blood (PaO_2_) to the inspired oxygen fraction (FiO_2_) (PaO_2_/FiO_2_), serum creatinine (sCr), blood urea nitrogen (BUN), lactate (Lac), and platelet (PLT) were collected to assess the cardiac function, respiratory function, renal function, and hematologic function. The data abstractors were blinded to the study objectives and hypothesis.

### 2.4. Statistical Analysis

Data were analyzed using SPSS (v.22.0) (SPSS Inc., Chicago, IL). Continuous variables were summarized as means ± standard derivations (SD) for normal distribution data and as median (interquartile range) for abnormal distribution data. All variables were tested for normal distribution by using the Kolmogorov–Smirnov test. Partial state distribution measurement data were transferred to normal distribution by the Ln treatment. Student's *t*-test was used to compare the means of continuous variables and normally distributed data; otherwise, the Mann–Whitney *U* test was used. The *chi*-square test was used to compare the categorical data. The risk factors were assessed by applying multivariable *Cox* regression analysis to obtain variables independently correlated with 28-day mortality in the pediatric patients with SALI. In order to appreciate the accuracy of independently risk factors as a prognostic marker, a ROC curve was generated. Survival curves were obtained by the Kaplan–Meier method and were compared by standard *log-rank* tests. A value of *P* < 0.05 was considered statistically significant.

## 3. Results

**Figure 1:**
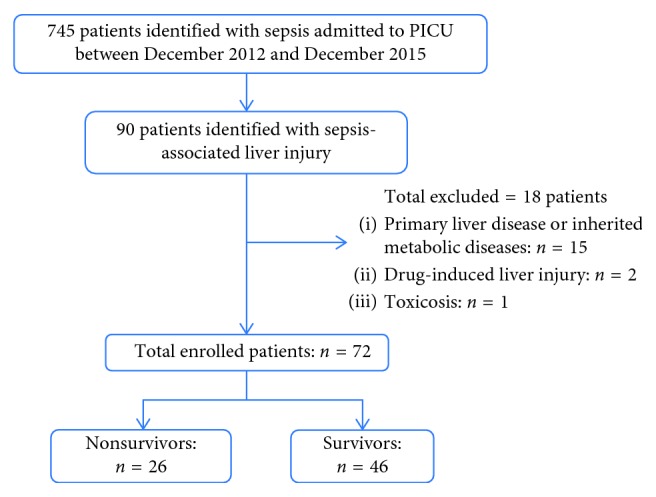
Flowchart for patient enrollment.

A total of 745 children diagnosed as sepsis were admitted to the PICU in Shanghai Children's Hospital between December 2012 and December 2015. Among them, a total of 90 patients were diagnosed as SALI. Fifteen patients were excluded due to primary liver disease and inherited metabolic diseases, 2 patients were excluded due to drug-induced liver injury, and 1 patient was excluded due to toxicosis. Finally, 72 patients with median age of 12 (7–48) months that met inclusion criteria were enrolled in this study, which included 43 male children and 29 female children (Figure 1). Forty-six patients were survived, and 26 patients were died. The mortality of SALI was 36.1% (26/72). The ratio of patients transferred from the other wards in survivor and nonsurvivor was similar (30.4% vs. 23.1%, *P*=0.503). The demographic and clinical characteristics of the patients are detailed in Table 1.

### 3.1. Baseline Characteristics and Classification of the Patients

The characteristics of the patients in the survivor group and nonsurvivor group were similar with respect to age, gender, source of sepsis, PRISM III, complications, and major pathogen (all *P* > 0.05). The levels of MAP, PaO_2_/FiO_2_, sCr, BUN, Lac, and PLT showed no significant difference in survivor compared with nonsurvivor with SALI (all *P* > 0.05). There was no significant difference about the rate of CRRT as adjuvant treatment between the two groups (*P* > 0.05), and the rate of mechanical ventilation was significantly higher in the nonsurvivor group (69.2%) compared with the survivor group (40.0%) (*P* < 0.05, Table 1).

### 3.2. Associations of Serum TBIL with the Outcome of Patients with SALI

The clinical characteristics were compared between the survivors and nonsurvivors to evaluate the risk factor for PICU mortality of the patients with SALI. The serum levels of TBIL, PT, APTT, and TBA were significantly higher in nonsurvivors with SALI compared with survivor with SALI (all *P* < 0.05) (Table 2). Multivariable *Cox* regression analysis was performed with these variables and showed that TBIL concentration was significantly associated with PICU mortality (HR 2.11, 95% CI: 1.14–3.88, *P*=0.017) (Table 3).

### 3.3. ROC Curve Analyses in Predicting the Outcome of Patients with SALI

A ROC curve was used to evaluate the variables as predictors for the prognosis in the pediatric patients with SALI. The area under the ROC curve (AUC) for PRISM III, PT, APTT, or TBA was 0.635 (0.497–0.773), 0.655 (0.524–0.786), 0.683 (0.551–0.815), or 0.662 (0.535–0.789), respectively, which were inferior to TBIL (AUC = 0.736, 95% CI: 0.614–0.858). Furthermore, the combined indexes including TBIL and PT (TBIL and PT), TBIL and APTT (TBIL and APTT), and TBIL and TBA (TBIL and TBA) were used to assess the power to predict the prognosis in patients with SALI. And the results indicated the AUCs for TBIL and PT, TBIL and APTT, and TBIL and TBA were 0.739 (0.618–0.861), 0.730 (0.608–0.851), and 0.745 (0.626–0.865), respectively (Table 4).

**Figure 2:**
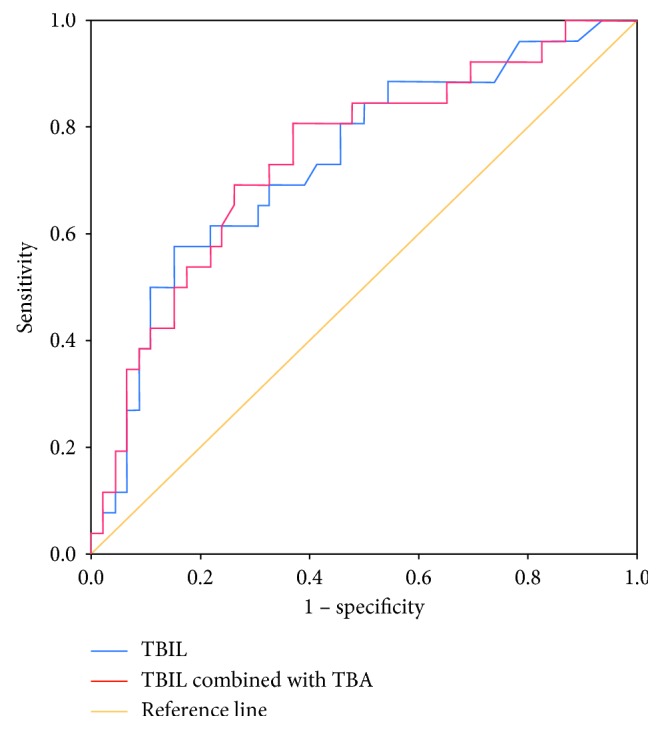
Receiver-operating characteristic (ROC) curves for the prognosis of the pediatric patients with SALI. TBIL is presented as a blue line, TBIL combined with TBA as a red line, and reference value as a brown line. The area under the ROC curve (AUC) for TBIL and TBIL combined with TBA is 0.736 (0.614–0.858) and 0.745 (0.626–0.865), respectively.

**Figure 3:**
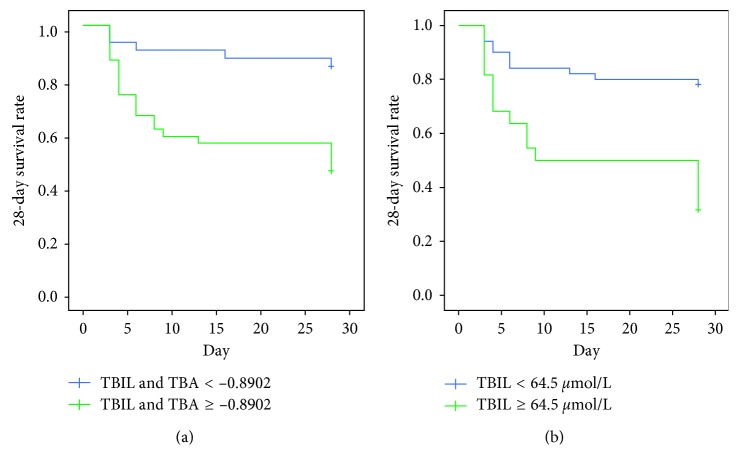
Kaplan–Meier curves for the patients with SALI: (a) serum TBIL ≥ 64.5 *μ*mol/L compared with serum TBIL < 64.5 *μ*mol/L; (b) TBIL combined with TBA (TBIL and TBA) ≥ −0.8902 compared with TBIL and TBA < −0.8902.

The optimal cutoff level was determined as the value capable of maximizing the prognostic accuracy. The optimal cutoff of 64.5 *μ*mol/L for serum TBIL concentration offered a sensitivity of 57.7% and specificity of 84.8%, and the optimal cutoff for the combined index of TBIL and TBA was −0.8902 (Figure 2). We further investigated the association between the serum TBIL level and the 28-day survival rate in pediatric patients with SALI using the Kaplan–Meier method. The pediatric patients with the higher serum TBIL level (≥64.5 *μ*mol/L) or TBIL and TBA (≥−0.8902) showed lower 28-day survival rate compared with the patients with the lower serum TBIL level (<64.5 *μ*mol/L) or TBIL and TBA (≤−0.8902) (Figure 3, *P* < 0.001). Therefore, the elevated serum TBIL level is associated with poor outcome of the pediatric patients with SALI.

## 4. Discussion

Liver dysfunction is common in patients with severe sepsis, and the prognosis of patients with SALI is unsatisfied [5, 14]. Therefore, it is urgently needed to identify a prognostic biomarker for the patients with SALI. In present study, we found that serum TBIL is an independent predictor for prognosis of the pediatric patients with SALI. The serum TBIL and combined index of TBIL and TBA showed better accuracy to predict prognosis in the pediatric patients with SALI. And serum TBIL ≥ 64.5 *μ*mol/L (3.77 mg/dl) implied high risk of poor outcome in the pediatric patients with SALI.

Liver dysfunction is recognized as one of the components that contribute to the severity of sepsis. Nevertheless, the incidence of liver dysfunction in the pediatric patients remains imprecise. The incidence of liver dysfunction in severe sepsis is estimated to be from 1% to 47%, depending on the restrictiveness of the definition [4]. In adult, “liver injury” was defined as a state in which the patient's blood laboratory results met at least one of four conditions: (i) serum TBIL of 3.0 mg/dl or greater; (ii) aspartate aminotransferase (AST) of 41 IU/L or greater; (iii) ALT of 41 IU/L or greater; and (iv) *γ*-GT of 51 IU/L or greater; and the incidence of SALI was 34.7% (156/449) [6]. In our present study, “liver injury” in the pediatric patients was defined by the following conditions: (1) TBIL ≥ 4.0 mg/dl or 68 *μ*mol/L or (2) ALT 2 times upper limit of normal for age [11]; and the incidence of SALI in the pediatric patients was 9.7% (72/745) in our study. In addition, the mortality of the adult patients with sepsis is 42% with serum TBIL > 2.0 mg/dl [10], and the mortality of the pediatric patients with SALI with serum TBIL > 4.0 mg/dl is 36.1% (26/72) in our present study. Based on our present study, the incidence and mortality of the pediatric patients with SALI is relatively lower than that of the adult patients. Given that age is variable affecting the assessment of liver dysfunction [15], we speculated that the differences of incidence and mortality of SALI between adult and children might be also related to the variable of age, besides of definition of SALI.

Liver injury affects a variety of metabolic pathways in the critically ill, but prognostic indicators of liver injury in the pediatric patients remain poorly understood. In present study, we found that the PT, APTT, TBA, and TBIL levels were higher in nonsurvivors than that of survivors, suggesting that blood coagulation function and bile acid metabolism might be related to the risk of death of patients with SALI. The liver plays a key role in blood coagulation function [16]. Accumulated evidences indicated the potential value of blood coagulation indicator as a prognostic marker. The initial antithrombin (AT) levels were significantly lower in newborns who died as compared with survivors, and lower initial AT levels in neonatal sepsis are associated with increased mortality, but not PT and APTT [17]. In our present study, the level of PT and APTT was higher in the pediatric patients with SALI who died as compared with survivors, but not associated with increased mortality. Whether the initial AT level in the pediatric patients is an independent predictor for outcome needs further investigation. Moreover, the plasminogen activator inhibitor 1 (PAI-1) promotes a poor prognosis in sepsis-induced disseminated intravascular coagulation [18]. And high plasma fibrinogen and low plasminogen are associated with poor survival in the chronic thromboembolic pulmonary hypertension (CTEPH) patients [19]. The potential value of all these blood coagulation indicator needs further study. In another aspect, liver dysfunction has been considered a prominent feature of the multiple organ dysfunction syndromes (MODS) and has been defined predominantly by hyperbilirubinemia and clinical jaundice [20, 21]. In this study, the higher serum TBIL and TBA levels in nonsurvivors of the pediatric patients implied that sepsis induces severe bile acid metabolic disorder or cholestasis contributing to liver injury. Furthermore, the combined index of TBIL and TBA showed a better power to assess the prognosis in patients with SALI comparing with single TBIL. Bile acids modulate innate and adaptive immunity and are involved in many metabolic and inflammatory processes mainly through activation of the nuclear receptor farnesoid *X* receptor (FXR), the membrane receptor TGR5, extracellular signal-regulated kinase (ERK), and MAP kinases signalling pathways [22]. It needs pay attention to the regulator of bile acid metabolism to find their potential prognostic value for the pediatric patients with SALI in the future.

Elevated serum TBIL suggesting poor liver function might intuitively be considered a marker of poor outcome. In present, our study indicated higher serum TBIL levels (≥64.5 *μ*mol/L) were closely associated with worse 28-day survival rate of patients with SALI. Consistently, Patel et al. [10] demonstrated that elevated serum TBIL levels (>2.0 mg/dL) within 72 hours after admission are associated with an increased risk of mortality in the adult patients with severe sepsis and septic shock. All these results suggested that serum TBIL might have universal significance for prognostic prediction in either adults or children with either SALI or sepsis. Furthermore, the optimal cutoff value of serum TBIL was 64.5 *μ*mol/L for prognostic prediction in the pediatric patients with SALI in the present study. In another clinical trial, the cutoff value of serum TBIL was 1.35 mg/dL for prognosis of the adult patients with prolonged sepsis [23]. The difference of the cutoff value for serum TBIL possibly relies on age and disease, which need further investigation through well-designed clinical trials.

Our study has some limitations. First, this is a retrospective study with a limited number of patients from a single center. Second, the data about the long-term mortality (for example, 90-day mortality) were not collected. If available, we would enable a more complete and robust analysis as to the risk of mortality in the future. Nevertheless, our results are noteworthy because the observation of serum TBLI as an independent risk factor for poor outcomes in the pediatric patients with SALI warrants further attention.

## 5. Conclusion

In summary, our study demonstrates that an elevated serum TBIL level within 72 hours after PICU admission is an independent risk factor of 28-day mortality in patients with SALI. Serum TBIL has a higher discriminatory power with a cutoff value as 64.5 *μ*mol/L to predict 28-day survival rate of the pediatric patients with SALI. Detection of serum TBIL may become a simple, convenient, and powerful clue for predicting prognosis in the pediatric patients with SALI.

## Figures and Tables

**Table 1:** Demographic and clinical characteristics of the patients enrolled in this study.

Characteristics	Survivor (*n*=46)	Nonsurvivor (*n*=26)	*P*
Age, month, median (IQR)	12 (7, 33)	18 (10, 84)	0.213
Male, *n* (%)	30 (65.2)	13 (50)	0.206
Source of sepsis			
Lungs, *n* (%)	25 (54.3)	11 (42.3)	0.326
Abdominal, *n* (%)	13 (28.3)	11 (42.3)	0.225
CNS, *n* (%)	4 (8.7)	1 (3.8)	0.437
Haematogenous, *n* (%)	2 (4.3)	2 (7.7)	0.552
Skin/soft tissues, *n* (%)	2 (4.3)	1 (3.8)	0.919
Pediatric risk of mortality III (PRISM III), median (IQR)	10 (5, 13)	12.5 (9, 19)	0.058
Complications			
Hemophagocytic syndrome, *n* (%)	7 (15.2)	5 (19.2)	0.661
Hematologic malignancy, *n* (%)	4 (8.7)	4 (15.4)	0.386
Adjuvant treatment			
CRRT, *n* (%)	13 (28.3)	6 (23.1)	0.632
Mechanical ventilation, *n* (%)	17 (40.0)	18 (69.2)	0.008
Transferred from the other ward, *n* (%)	14 (30.4)	6 (23.1)	0.503
MAP^*∗*^ (mmHg)	67 (61–73)	66 (57–76)	0.518
PaO_2_/FiO_2_^*∗*^ (mmHg)	331 (241–410)	300 (203–365)	0.078
sCr^*∗*^ (*μ*mol/L)	26.5 (20.0–43.0)	31.5 (22.0–48.0)	0.443
BUN^*∗*^ (mmol/L)	4.2 (2.9–7.1)	5.5 (3.2–11.0)	0.212
Lac^*∗*^ (mg/dL)	2.3 (1.3–3.6)	2.7 (1.4–5.2)	0.303
PLT^*∗*^(^*∗*^109/L)	183 (119–333)	157 (57–208)	0.199
Major pathogen			
Gram-negative bacterium	22	13	0.859
*Acinetobacter baumannii*	7	5	
*Pseudomonas aeruginosa*	5	2	
*Klebsiella pneumoniae*	3	3	
*Haemophilus influenzae*	4	0	
*Enterobacter cloacae*	0	2	
Others	3	1	
Virus	14	4	0.157
EBV	11	3	
CMV	3	1	
Gram-positive bacterium	8	4	0.826
*Streptococcus pneumoniae*	1	2	
*Staphylococcus aureus*	2	1	
*Enterococcus gallinarum*	2	0	
*Enterococcus faecium*	1	1	
*Staphylococcus capitis*	1	0	
Others	1	0	
Mycoplasma	2	1	0.919

CNS: central nervous system; CRRT: continuous renal replacement therapy; MAP: mean arterial pressure; PaO_2_/FiO_2_: the ratio of the partial pressure of oxygen in arterial blood (PaO_2_) to the inspired oxygen fraction (FiO_2_); sCr: serum creatinine; BUN: blood urea nitrogen; Lac: lactate; PLT: platelet. ^*∗*^Indicated that data were treated with Ln processing.

**Table 2:** Biochemical parameters of the patients enrolled in this study.

Parameter	Survivor (*n*=46)	Nonsurvivor (*n*=26)	*P*
ALT^*∗*^ (U/L)	224 (127–465)	239 (108–405)	0.862
*γ*-GT^*∗*^ (U/L)	70 (35–227)	70 (30–279)	0.872
TBIL^*∗*^ (*μ*mol/L)	13 (7–46)	73 (21–130)	0.001
ALB (g/L)	32 (29–37)	29 (26–33)	0.152
PT^*∗*^ (s)	13.9 (12.0–16.1)	15.4 (13.0–19.1)	0.023
APTT^*∗*^ (s)	36.4 (29.9–42.1)	44.0 (34.9–58.0)	0.002
TBA^*∗*^ (*μ*mol/L)	20 (8–54)	47 (32–96)	0.021
INR^*∗*^	1.20 (1.03–1.41)	1.28 (1.11–1.55)	0.104

ALT: alanine transaminase; *γ*-GT: *γ*-glutamyltransferase; TBIL: total bilirubin; ALB: albumin; PT: prothrombin time; APTT: activated partial thromboplastin time; INR: international normalized ratio; TBA: total bile acid. Data are presented as median (IQR). ^*∗*^Indicated that data were treated with Ln processing.

**Table 3:** Predictive capacity for PICU mortality of the selected variables in patients with SALI.

Variables	HR	95% CI	*P*
TBIL	2.11	1.14–3.88	0.017
PT	1.97	0.42–9.33	0.392
APTT	2.57	0.62–10.59	0.192
TBA	0.61	0.31–1.18	0.140

TBIL: total bilirubin; PT: prothrombin time; APTT: activated partial thromboplastin time; TBA: total bile acid; HR: hazard ratio; 95% CI: 95% confidence interval.

**Table 4:** Values of variables for predicting the prognosis in patients with SALI using ROC analysis.

Variables	AUC	95% CI	*P*
PRISM III	0.635	0.497–0.773	0.059
TBIL	0.736	0.614–0.858	0.001
PT	0.655	0.524–0.786	0.030
APTT	0.683	0.551–0.815	0.010
TBA	0.662	0.535–0.789	0.023
TBIL and PT	0.739	0.618–0.861	0.001
TBIL and APTT	0.730	0.608–0.851	0.001
TBIL and TBA	0.745	0.626–0.865	0.001

PRISM III: pediatric risk of mortality III; TBIL: total bilirubin; PT: prothrombin time; APTT: activated partial thromboplastin time; TBA: total bile acid.

## Data Availability

The data used to support the findings of this study are available from the corresponding author upon request.
